# The tuberculosis necrotizing toxin is an NAD^+^ and NADP^+^ glycohydrolase with distinct enzymatic properties

**DOI:** 10.1074/jbc.RA118.005832

**Published:** 2018-12-28

**Authors:** Uday Tak, Jiri Vlach, Acely Garza-Garcia, Doreen William, Olga Danilchanka, Luiz Pedro Sório de Carvalho, Jamil S. Saad, Michael Niederweis

**Affiliations:** From the ‡ Department of Microbiology, University of Alabama at Birmingham, Birmingham, Alabama 35205 and; the § Francis Crick Institute, London NW1 1AT, United Kingdom

**Keywords:** bacterial toxin, tuberculosis, enzyme kinetics, enzyme mechanism, host-pathogen interaction

## Abstract

Upon host infection, *Mycobacterium tuberculosis* secretes the tuberculosis necrotizing toxin (TNT) into the cytosol of infected macrophages, leading to host cell death by necroptosis. TNT hydrolyzes NAD^+^ in the absence of any exogenous cofactor, thus classifying it as a β-NAD^+^ glycohydrolase. However, TNT lacks sequence similarity with other NAD^+^ hydrolyzing enzymes and lacks the essential motifs involved in NAD^+^ binding and hydrolysis by these enzymes. In this study, we used NMR to examine the enzymatic activity of TNT and found that TNT hydrolyzes NADP^+^ as fast as NAD^+^ but does not cleave the corresponding reduced dinucleotides. This activity of TNT was not inhibited by ADP-ribose or nicotinamide, indicating low affinity of TNT for these reaction products. A selection assay for nontoxic TNT variants in *Escherichia coli* identified four of six residues in the predicted NAD^+^-binding pocket and four glycine residues that form a cradle directly below the NAD^+^-binding site, a conserved feature in the TNT protein family. Site-directed mutagenesis of residues near the predicted NAD^+^-binding site revealed that Phe^727^, Arg^757^, and Arg^780^ are essential for NAD^+^ hydrolysis by TNT. These results identify the NAD^+^-binding site of TNT. Our findings also show that TNT is an NAD^+^ glycohydrolase with properties distinct from those of other bacterial glycohydrolases. Because many of these residues are conserved within the TNT family, our findings provide insights into understanding the function of the >300 TNT homologs.

## Introduction

**Figure 1.**
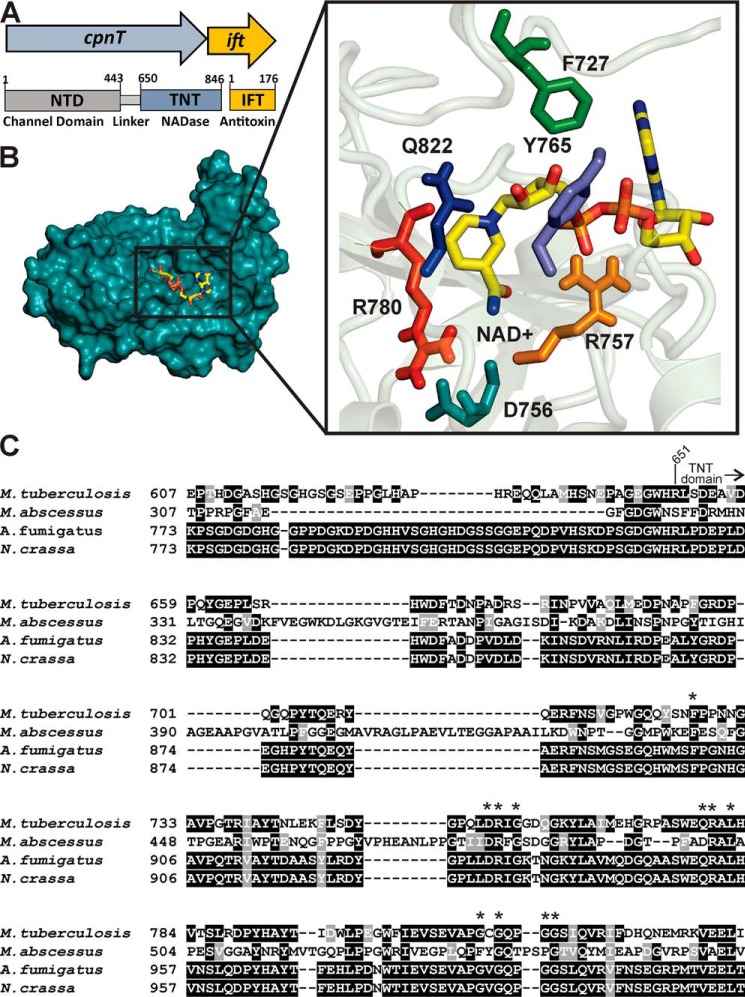
**TNT family enzymes contain conserved catalytic residues.** *A*, the *cpnT–ift* operon of *M. tuberculosis* and the domain organization of CpnT. *B*, putative NAD^+^-binding site of TNT in the docking model (18) with conserved residues highlighted. *C*, alignment of TNT homologs in mycobacteria and fungi. All sequences were obtained from Pfam: Pf14021. The sequence alignment was visualized using the T-Coffee and BoxShade servers.

Since the discovery of diphtheria toxin in 1888 (1), over 300 bacterial toxins have been identified, many of which have been established as the causative agents of diseases (2–4). These toxins are often secreted and subvert and/or damage host cells by a wide variety of mechanisms including covalent modification of target proteins (5, 6), pore formation (7), protein degradation (8), and others (9–12). Toxins with ADP-ribosyltransferase activity that cleave NAD^+^ and attach the hydrolysis product ADP-ribose onto a target protein are widespread in pathogenic bacteria (5, 6). Only a few bacterial toxins have strict NAD^+^ glycohydrolase activity and appear to kill host cells by depleting NAD^+^ in the absence of an exogenous target. The best-studied example is the *Streptococcus pyogenes* NAD^+^ glycohydrolase SPN, which is secreted together with streptolysin O (13). Streptolysin O forms membrane pores and mediates translocation of SPN into target cells (14, 15). The NAD(P)^+^ glycohydrolase Tse6 of *Pseudomonas aeruginosa* is injected into other bacteria via a type VI secretion system and kills by NAD(P)^+^ depletion (16). Recently, we have shown that the outer membrane protein CpnT of *Mycobacterium tuberculosis* consists of an N-terminal outer membrane channel (17) and a C-terminal NAD^+^-glycohydrolase (Fig. 1*A*) (18). The NAD^+^ glycohydrolase domain (TNT)^5^ is secreted by *M. tuberculosis* and gains access to the cytosol of macrophages infected with *M. tuberculosis* following phagosome rupture (17, 18). The glycohydrolase activity of TNT is required for the survival of *M. tuberculosis* in macrophages (18), and the subsequent NAD^+^ depletion activates the necroptosis pathway in host cells (19). Although TNT promotes intracellular survival and replication of *M. tuberculosis*, a large family of secreted NAD^+^ glycohydrolases with similarities to TNT was shown to be involved in interbacterial competition in Firmicutes (20, 21). An important distinction between NAD^+^ glycohydrolases and ADP-ribosyltransferases is that the latter enzymes hydrolyze NAD^+^ with very low turnover numbers of less than 0.17 s^−1^ in the absence of their target proteins. In contrast, glycohydrolases such as SPN and Tse6 rapidly cleave NAD^+^ with rates of 8390 and 1983 s^−1^, respectively (13, 16). Interestingly, SPN shares sequence similarities with both ADP-ribosyl cyclases, which produce cyclic ADP-ribose as a by-product, and ADP-ribosyltransferases including the ARTT motif with the catalytically essential glutamate residue Glu^391^ (13). The structure of TNT is distinct from those of SPN and ADP-ribosyltransferases because it lacks the ARTT and other motifs involved in NAD^+^ binding and hydrolysis by these enzymes (18, 22) (Fig. S1), suggesting a different catalytic mechanism. The goal of this study was to examine the enzymatic properties of TNT using random mutagenesis and genetic selection and structure-guided approaches. We characterized the substrate specificity and the kinetic activity of TNT. Further, we identified catalytic residues of TNT that are different from those required for the enzymatic activity of SPN and ADP-ribosyltransferases. The molecular model derived from these results provides a better understanding of substrate hydrolysis by TNT. This model may also help to examine the functions of the more than 300 homologs in the TNT protein family, previously known as the DUF4237 domain.

## Results

### TNT hydrolyzes NAD^+^ and NADP^+^ but not their corresponding reduced forms

**Figure 2.**
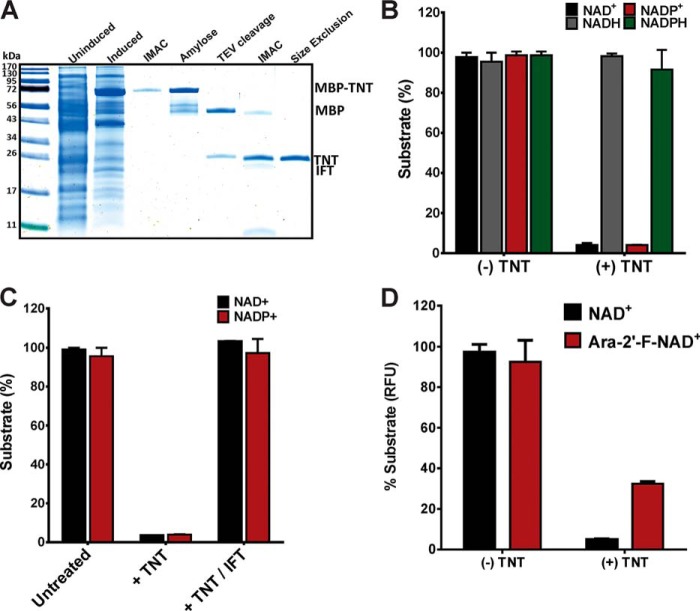
**Purification and substrate specificity of TNT of *M. tuberculosis*.** *A*, colloidal Coomassie-stained SDS-polyacrylamide gel samples for each step during purification of TNT. The lanes (*left* to *right*) show the protein marker, uninduced starter culture, autoinduced whole cell lysate, immobilized nickel affinity chromatography (*IMAC*), amylose affinity chromatography, TEV protease cleavage, IMAC removal of TEV protease and MBP, and size exclusion on a Superdex 75 column. *B*, substrate hydrolysis by TNT was measured using enzymatic assays at a protein concentration of 75 nm and a substrate concentration of 100 μm at 37 °C and pH 7.0 for 30 min. The substrate concentrations for the samples without TNT were set to 100%. The substrate concentrations in the samples with TNT were normalized compared with the untreated sample. *C*, inhibition of NAD(P)^+^ hydrolysis by TNT in the presence of IFT using the same conditions as in *B. D*, hydrolysis of araF-NAD^+^ by TNT using the same assay and conditions as in *B*. The experiments were performed twice with similar results. The standard deviations are shown as *error bars*.

Although ADP-ribosyltransferases cleave only NAD^+^, bacterial glycohydrolases cleave both NAD^+^ and NADP^+^. TNT was shown to hydrolyze NAD^+^ (18), but the activity of TNT toward other dinucleotides was not investigated. This was partly due to the difficulties in purification of TNT, which must be co-expressed with its antitoxin IFT and separated by heat, resulting in a loss of over 70% target protein (18). To this end, we improved the purification protocol of TNT by replacing the heat-denaturation step (18) by treatment with 8 m urea or 4 m guanidine hydrochloride to remove the inhibitor IFT from the His_6_–MBP–TNT fusion protein. TNT was then refolded on a column and purified by standard affinity chromatography techniques (Fig. 2*A*). The His_6_–MBP–TNT protein was then purified by amylose affinity chromatography and, after TEV protease cleavage and removal of the affinity tag, by size-exclusion chromatography (Fig. 2*A*). Using this protocol, we obtained ∼0.25 mg of pure TNT protein per liter of *Escherichia coli* culture (Fig. 2*A*).

Next, we investigated the substrate specificity of TNT using a fluorescence-based enzyme cycling assay as previously described (18). TNT hydrolyzed the dinucleotides NAD^+^ and NADP^+^ but not their reduced forms NADH and NADPH (Fig. 2*B*). Addition of the antitoxin IFT prevented hydrolysis of both NAD^+^ and NADP^+^ (Fig. 2*C*). This result indicates that both dinucleotides are cleaved at the same site because IFT blocks access to a cleft predicted as the NAD^+^-binding site by docking modeling (18).

To further characterize the catalytic activity of TNT, we tested the ability of TNT to cleave the NAD^+^ analog β-ara-2′-deoxy-2′-fluoro-nicotinamide adenine dinucleotide (araF-NAD^+^), which acts as a slow binding inhibitor of eukaryotic NAD^+^ hydrolases (23). araF-NAD^+^ was cleaved by TNT, albeit more slowly than NAD^+^ (Fig. 2*D*).

### TNT does not produce cyclic ADP-ribose

Some NAD^+^-hydrolyzing enzymes produce cyclic ADP-ribose as a side product in addition to nicotinamide and ADP-ribose (13). A complete assignment of all ^1^H NMR peaks for NAD^+^, nicotinamide, ADP-ribose, and cyclic ADP-ribose did not reveal any signals specific for cyclic ADP-ribose after hydrolysis of NAD^+^ by TNT (Fig. S2). These results show that TNT does not produce any cyclic ADP-ribose detectable under those conditions.

### Direct determination of kinetic parameters of NAD^+^ and NADP^+^ hydrolysis by TNT

**Figure 3.**
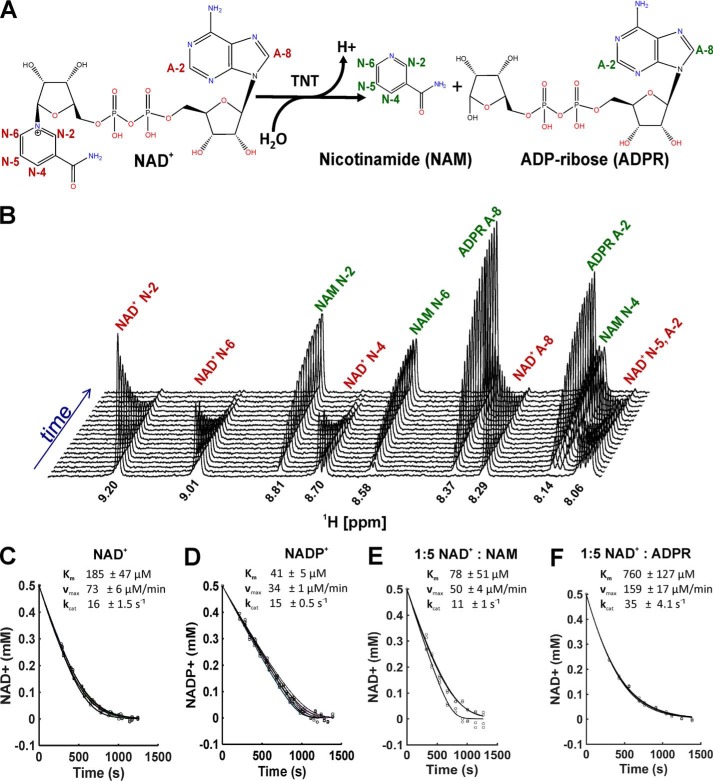
**Kinetic analysis of TNT substrate hydrolysis and inhibition.** *A*, NAD^+^ hydrolysis by TNT. *N* and *A* indicate protons from nicotinamide and adenine, respectively. *Red* indicates signals corresponding to NAD^+^, whereas *green* represents signals from hydrolyzed products. *B*, time course of NAD^+^ hydrolysis and nicotinamide and ADP-ribose formation as followed by ^1^H NMR. Chemical shifts and assignments of the signals are indicated. *C*, NAD^+^ (500 μm) hydrolysis by 75 nm TNT and average kinetic parameters (*n* = 3). The signal intensities corresponding to NAD^+^ N-2, N-4, and N-6 were plotted and used for curve fitting in *C* and *D. D*, NADP^+^ (500 μm) hydrolysis by 37.5 nm TNT and average kinetic parameters (*n* = 3). *E*, NAD^+^ (500 μm) hydrolysis kinetics in the presence of 75 nm TNT and 2.5 mm nicotinamide (*n* = 2). The signal intensities corresponding to NAD^+^ N-2 and N-6 were plotted and used for curve fitting in *E* and *F. F*, NAD^+^ (500 μm) hydrolysis kinetics in the presence of 75 nm TNT and 2.5 mm ADP-ribose (*n* = 3).

We used ^1^H NMR to directly measure the kinetics of TNT-mediated hydrolysis of NAD^+^ and NADP^+^. To this end, we followed the resonance decay of NAD^+^ protons and the resonance increase of nicotinamide and ADP-ribose protons (Fig. 3*A*). An advantage of NMR is that it directly and quantitatively records substrate hydrolysis and product accumulation in real-time over the entire course of the reaction (Fig. 3*B*) as shown previously (24). The Michaelis constant *K_m_* of ∼190 ± 50 μm and the turnover rate *k*_cat_ of 16/s for NAD^+^ hydrolysis by TNT were determined using a direct numerical solution of the Michaelis–Menten equation. Both substrate-binding affinity and maximal NAD^+^ hydrolysis rates measured by NMR were slightly different compared with previous values (Ref. 18 and Table 1). This difference could be due to the different protein purification methods, the different methods of NAD^+^ detection, and/or the different buffer conditions. Because the NMR method detects NAD^+^ hydrolysis directly and enables the measurement of kinetics continuously and more reproducibly, we used NMR in subsequent experiments to examine the catalytic properties of TNT. Using NMR, we showed that TNT hydrolyzed NAD^+^ and NADP^+^ with similar specificity constants in contrast to SPN, which had a 20-fold reduced rate for NADP^+^ cleavage (Table 1). Our results are consistent with a *K_m_* of 310 μm determined previously for NAD^+^ hydrolysis in *M. tuberculosis* extracts (25).

**Table 1:** **Kinetic properties of NAD^+^-hydrolyzing enzymes** The bacterial NAD(P)^+^ glycohydrolases include TNT of *M. tuberculosis*, SPN of *S. pyogenes*, and Tse6 of *P. aeruginosa*. The values for TNT were determined in this study unless otherwise indicated. The cholera toxin was taken as an example for the enzymatic properties of ADP-ribosyltransferases (ADP-RTs), whereas CD38 served as an example for multifunctional NADases. Note that the value for ADP-RTs is the NAD^+^ glycohydrolase activity in the absence of target proteins.

Enzymatic properties	Bacterial glycohydrolases			ADP-RT	Multifunctional
	TNT	SPN	Tse6	Cholera toxin	CD38
*K_m_* (NAD^+^) [μm]	190 ± 50	188 ± 23 (13)	N/A	4000–14,000 (52, 53)	46 ± 4 (28)
*k*_cat_ (NAD^+^) [s^−1^]	16 ± 1.5	8390 (13)	1983 (16)	<10 (13)	148 ± 8 (13)
*K_m_* (NADP^+^) [μm]	41 ± 1	1.7 ± 0.4 (13)	N/A	N/A	65 ± 19 (28)
*k*_cat_ (NADP^+^) [s^−1^]	15 ± 0.5	384 (13)	983 (16)	N/A	3.3 × 10^−5^ (28)
Nicotinamide inhibition	No	No (13)	?	Yes	Yes (54)
ADP-ribose inhibition	No	Yes (13)	?	No	Yes (28)
cADPR generation	No (18)	No (13)	?	No (13)	Yes (28)
ADP-ribosylation	No (18)	No (13)	No (16)	Yes (13)	Yes (28, 54)
β-NAD^+^ methanolysis	No	No (13)	?	No (52)	Yes (54)

### TNT is not inhibited by the hydrolysis products nicotinamide and ADP-ribose

Some enzymes are inhibited by their reaction products as a physiological feedback mechanism to regulate enzymatic activity. This includes the NAD^+^ glycohydrolases SPN, ADP-ribosyltransferases, and mammalian CD38, whose activities are inhibited by ADP-ribose alone or by ADP-ribose and nicotinamide, respectively (13). To determine whether product inhibition regulates TNT activity, we performed NAD^+^ hydrolysis experiments in the presence of increasing quantities of nicotinamide and ADP-ribose. We did not observe a decrease in the NAD^+^ hydrolysis rate in the presence of ADP-ribose or nicotinamide at a 5-fold molar excess over NAD^+^ by NMR (Fig. 3, *E* and *F*) or reverse-phase HPLC (not shown). These results are consistent with the proposed function of TNT, which would be self-limiting if product inhibition was present.

### TNT does not cleave NAD^+^ using methanol as a nucleophile

**Figure 4.**
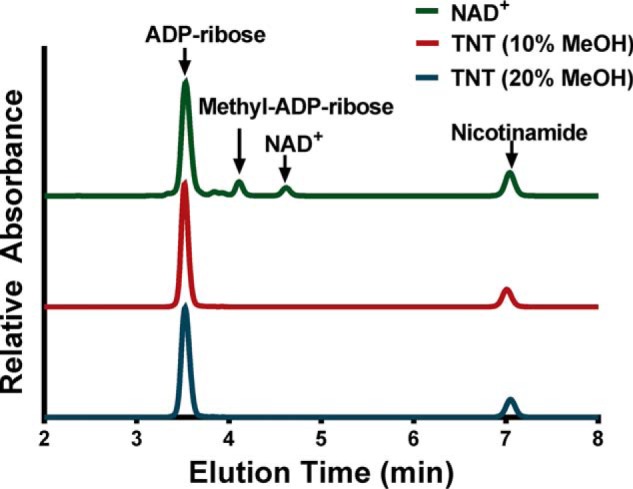
**TNT does not catalyze methanolysis.** Reverse-phase HPLC chromatogram displaying the reaction products obtained from nonenzymatic methanolysis of β-NAD^+^ (*green*) and those from TNT-mediated NAD^+^ hydrolysis (*red* and *blue*). 29 nm TNT was used for this experiment. The absorbance was measured at 260 nm.

NAD^+^-hydrolyzing enzymes are often classified in three groups depending on the stereochemical outcome of the reaction they catalyze, *i.e.* retention of the β-anomeric configuration, inversion of configuration, or inability to form a configurationally stable product (26, 27). Analysis of the product configuration after methanolysis, a reaction in which methanol replaces water as the nucleophile, generating 1′-*O*-methyl ADP-ribose instead of ADP-ribose (26), would enable us to identify the stereochemistry of NAD^+^ cleavage by TNT because of the lower rate of isomerization of the product. Thus far, only mammalian NADases such as CD38 (28) and ADP-ribosyl cyclases (26) have been found to catalyze NAD^+^ methanolysis, whereas ADP-ribosyl transferases and nicotinamide-insensitive NADases do not (13). Because the ratio of hydrolysis *versus* methanolysis of NAD^+^ corresponds to the molar ratio of water to methanol in the reaction mixture (29), spontaneous cleavage of NAD^+^ in a water/methanol mixture was used as a positive control for formation of 1′-*O*-methyl ADP-ribose (Fig. 4). Using an HPLC-based assay, we detected methyl-ADP-ribose during spontaneous cleavage of NAD^+^ in the presence of up to 20% methanol, but not in the TNT-catalyzed cleavage of NAD^+^ (Fig. 4). These results indicate that TNT catalyzes hydrolysis but not methanolysis of NAD^+^.

### Random mutagenesis assay to identify residues critical for NAD^+^ hydrolysis by TNT

TNT has over 300 homologs in more than 250 bacterial and fungal species. This protein family (previously DUF4237) was recently named the TNT family in the Pfam database (PF14021). TNT homologs share residues that are distinct from SPN and ADP ribosyltransferases (Fig. 1*C* and Fig. S1). Two residues in the putative NAD^+^-binding cleft, Tyr^765^ and Gln^822^, which are similarly positioned and are essential in the diphtheria toxin, were shown to be important but not essential for TNT activity (18). Because of the very low rate of NAD^+^ hydrolysis in the absence of their ADP-ribosylation target, the mechanism of NAD^+^ binding and hydrolysis has been extensively studied in the diphtheria and cholera toxins by co-crystallization with NAD^+^ (14–16). In contrast, the rapid hydrolysis of NAD^+^ by glycohydrolases prevents the identification of NAD^+^-binding residues by X-ray crystallography.

**Figure 5.**
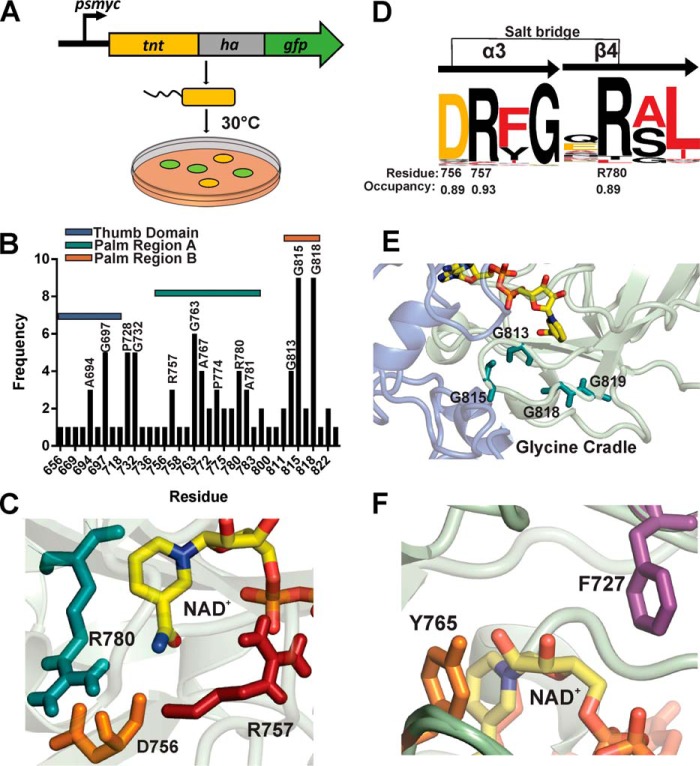
**Selection of nontoxic TNT mutants in *E. coli*.** *A*, scheme of the reporter selection screen. *Green* colonies indicate *E. coli* clones expressing full-length *tnt–gfp* mutants with reduced toxicity. *B*, number of mutants obtained at the indicated residue and the relative location within the TNT structure. *C*, potential DR*X*G–QR*X*L salt bridge in the predicted NAD^+^-binding site. *D*, the relative occupancy and residue number of the DR*X*G and QR*X*L motifs in CpnT obtained from the hidden Markov model using the TNT family of proteins and modeled using Skylign and WebLogo. *E*, glycine cradle located at the base of the NAD^+^-binding site predicted by the docking model. *F*, Tyr^765^ and Phe^727^ are located close to the nicotinamide moiety in the predicted NAD^+^-binding site.

To circumvent the challenges of the structural analysis of a TNT–NAD^+^ complex, we developed a genetic selection assay as an alternative approach to identify residues critical for the enzymatic activity of TNT. We hypothesized that only nontoxic TNT mutants would yield colonies on agar plates as sustained expression of WT TNT is only possible in *E. coli* in the presence of the antitoxin IFT (18). To this end, we constructed a transcriptional fusion of the *tnt* gene with the codon-optimized *gfp_m_*^2+^ (30) containing the mycobacterial p_smyc_ promoter in the expression plasmid pML2123 (Fig. 5*A* and Table S2). Then error-prone PCR was used to amplify the *tnt–gfp* DNA fragment and to introduce *tnt* mutations. Next, the library of mutated *tnt–gfp* genes in the expression plasmid pML2123 was transformed into *E. coli*. Green fluorescence indicated that the clones produced a functional TNT–GFP protein. The plasmids of 108 fluorescent *E. coli* clones were isolated and sequenced. We identified 94 TNT mutants with single-point mutations, of which 55 were nonredundant (Fig. S3). In addition, we obtained one triple mutant, seven double mutants, and six mutants with premature stop codons. The majority of the mutations were observed in two regions comprising residues 752–784 (region A) and 811–822 (region B). Region A contains many residues in the predicted NAD^+^-binding site (Figs. 1*B* and 5*B*), supporting our docking model of the TNT–NAD^+^ complex (18). Examples include the DR*X*G motif (Asp^756^, Arg^757^, and Gly^759^; Fig. 5, *C* and *D*) and the QR*X*L motif (Arg^780^), which are conserved in the TNT protein family (Fig. 1*C* and 5*C*). Surprisingly, half of the single-point TNT mutants contained mutated glycine residues (47). Many of those glycine residues are clustered in a loop comprising residues 811–822 (region B). In particular, mutations of the three glycines at positions 813 (4), 815 (9), and 818 (9) accounted for more than a quarter of all isolated TNT mutants. Gly^818^ is highly conserved in the TNT protein family (Fig. 1) and was mutated to valine in nine clones (Fig. S3). These glycines form a cradle, which is located below the putative active site (Fig. 5*E*). Interestingly, Pro^728^ was mutated twice but not the preceding highly conserved Phe^727^ (Fig. 5*F* and Fig. S3). Taken together, these results identify residues important for the catalytic activity and/or structural integrity of TNT.

### Structure-guided identification of residues involved in hydrolysis of NAD^+^ by TNT

TNT protein was detected in all but two *E. coli* clones expressing mutated *tnt–gfp* fusions in immunoblots using an anti-TNT antibody (not shown). The TNT–GFP fusion protein levels among the clones were different, indicating either protein folding and/or stability issues and/or residual toxicity. The latter was clearly observed by the apparent growth defect of a few selected *E. coli* clones. To further study residues involved in ligand binding and/or catalysis, we designed TNT constructs with mutations in the NAD^+^-binding site suggested by docking modeling. Two residues in this site, Gln^822^ and Tyr^765^ were previously shown to reduce the enzymatic activity of TNT providing experimental support for the model (18). Based on the proximity to NAD^+^, we mutated the residues Phe^727^, Asn^731^, Asp^756^, Arg^757^, Gln^762^, Arg^780^, and His^783^ to alanine in a *tnt* overexpression vector. To avoid the complications of potential residual toxicity, which could result in different protein levels, we produced and purified these TNT mutants in an *E. coli* strain, which co-produced the antitoxin IFT as described above for WT TNT (Fig. S4).

**Figure 6.**
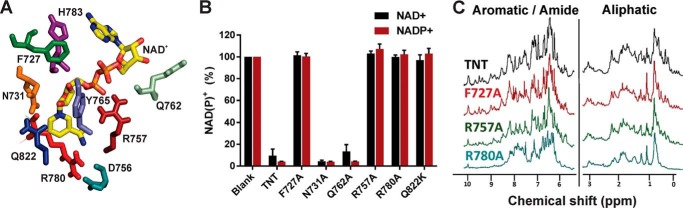
**Identification of the NAD^+^-binding site of TNT.** *A*, a docking model of the TNT–NAD^+^ complex and putative catalytic residues. *B*, NAD(P)^+^ hydrolysis by TNT and mutant proteins. Substrate hydrolysis by TNT was measured using enzymatic assays at a protein concentration of 75 nm and a substrate concentration of 100 μm at 37 °C and pH 7.0 for 30 min. Substrate concentrations in the samples without TNT were set to 100%, and those with TNT were normalized compared with the untreated sample. Each experiment was performed at least twice with similar results. Standard deviations are shown as *error bars. C*, ^1^H NMR spectra of 20 μm WT TNT and TNT mutants in 25 mm sodium phosphate, 150 mm NaCl, pH 7.0, with 5% D_2_O.

As shown in Fig. 5*C*, Asp^756^, Arg^757^, and Arg^780^ reside at the base of the active site and are predicted to bind NAD^+^. Arg^757^ is predicted to contact the phosphate backbone of NAD^+^, whereas Asp^756^ and Arg^780^ appear to form a salt bridge stabilizing the active site and bridging the DR*X*G and QR*X*L motifs (Fig. 5, *C* and *D*). TNT variants with mutations in these positions were obtained in the genetic selection assay, indicating the importance of these residues for toxicity of TNT (Fig. S3). Indeed, the purified R757A and R780A TNT proteins had no detectable NAD^+^ or NADP^+^ hydrolysis activity (Fig. 6*B*). To examine whether the lack of activity of these TNT mutants was due to a disruption of the TNT structure, we obtained ^1^H NMR spectra of the purified proteins to assess folding. The ^1^H NMR spectra of WT TNT and the R757A mutant protein were very similar, indicating that the mutation did not alter the overall structure of TNT (Fig. 6*C*). In contrast, a spectrum of different appearance with broader or missing peaks in the aliphatic, aromatic, and amide regions was obtained for the R780A mutant, indicating that the protein was partially unfolded (Fig. 6*C*). The Asp^756^ mutation resulted in an unstable protein, which precipitated immediately upon cleavage from the MBP-fusion protein (not shown), thus precluding further analysis. The instability of the D756A and the R780A mutant proteins indicates the importance of the salt bridge formed by these residues for the overall structure of TNT.

Phe^727^ was chosen because of its conservation in the TNT protein family (Fig. 1) and its location close to the nicotinamide moiety of NAD^+^ suggesting π-stacking (Fig. 5*F*). His^783^ was chosen because of its proximity to the adenine moiety and its selection via the reporter screen. The H783A protein precipitated immediately upon cleavage from MBP, similarly to the D756A mutant, indicating that His^783^ is a structurally important residue (not shown). By contrast, mutation of Phe^727^ to alanine completely disrupted the catalytic activity of TNT (Fig. 6*B*), apparently without affecting the structure of TNT (Fig. 6*C*). Furthermore, the F727A mutant protein was the most stable TNT mutant and produced the highest yield in *E. coli* (∼6 mg/liter culture) (Fig. S4).

We previously noticed that Tyr^765^ flanks the 2′-OH of nicotinamide-ribose but observed residual activity upon mutation to alanine (18). Thus, we hypothesized that Asn^731^, because of its proximity to Tyr^765^ (Fig. 6*A*), might also play a role in the stabilization of the 2′-OH group, a function that has been described as critical for hydrolysis of NAD^+^ (26). Gln^762^, located at the outer rim of the binding cleft, may also stabilize the phosphate groups on NADP^+^ and thereby distinguish between NAD^+^ and NADP^+^. However, we found that mutation of Asn^731^ and Gln^762^ to alanine did not affect NAD(P)^+^ hydrolysis by TNT (Fig. 6*B*), indicating that these mutations are dispensable for activity.

## Discussion

### Substrate specificity of TNT

In this study, we used an NMR assay to directly measure hydrolysis of NAD^+^ and NADP^+^ by TNT. The lower *K_m_* value for NADP^+^ compared with NAD^+^ suggests that NADP^+^ is a preferred substrate of TNT. This result is in contrast to the glycohydrolase SPN of *S. pyogenes*, which is 20-fold less efficient at hydrolyzing NADP^+^ compared with NAD^+^ (13). However, the observation that the reduced nucleotides, NADH and NADPH, are not substrates of TNT is similar to other glycohydrolases such as SPN. The absence of NADH hydrolysis by TNT is likely caused by the substantial structural changes of the nicotinamide ring, which adopts a puckered conformation after reduction (31). These structural differences can lead to binding affinities varying by more than 100-fold in NAD^+^/NADH-binding proteins (32). Additionally, the absence of a positive charge in NADH would prevent the formation of a putative oxocarbenium intermediate, which relies on the neutralization of the nicotinamide charge during the transition state (33). However, the absence of NADH hydrolysis by TNT in our experiments could be due to either lack of binding or lack of catalytic cleavage of NADH. Further experiments would be needed to distinguish between these possibilities.

### Inhibition and stereochemistry of NAD^+^ hydrolysis by TNT

In this study, we have shown that TNT is not inhibited by nicotinamide and ADP-ribose at 5-fold molar excess. This is consistent with the original report of the NADase activity in *M. tuberculosis*, which did not identify product inhibition (25). By contrast, SPN and bacterial ADP-ribosyltransferases such as the *P. aeruginosa* ExoS toxin are inhibited by ADP-ribose (13, 34). These differences may be reflected in the size of these proteins. Although TNT is relatively small (197 amino acids) and only has the minimally required elements for hydrolyzing NAD(P)^+^, SPN (451 amino acids) and ADP-ribosyltransferase toxins are much larger and have additional domains required for membrane or chaperone binding (2), which may be amenable to product inhibition or auto-ADP-ribosylation. We conclude that TNT is not regulated by hydrolysis products and will continuously hydrolyze available NAD^+^ or NADP^+^ as long as substrate is available.

### Identification of nontoxic TNT variants

Random mutagenesis of TNT in combination with a selection for nontoxic mutants yielded 55 single-point mutants. Four of the six residues (Asp^756^, Arg^757^, Arg^780^, and Gln^822^) predicted to form the NAD^+^-binding pocket by the docking model (Fig. 1*B* and Fig. S3*B*) were mutated, providing experimental evidence in support of the model (18). Altogether, 23 of 50 residues highly conserved in the TNT family (Fig. S3*B*) were isolated in our selection approach. This indicated the importance of the conserved residues for the function of TNT and related toxins. It is possible that mutations of the other half of the conserved residues that were not isolated in our genetic selection assay did not reduce the toxicity of TNT to the very low levels required for *E. coli* to survive. An alternative explanation is that these mutants were not present in our initial plasmid library.

The most surprising result of the selection assay was the finding that more than 25% of the mutated TNT residues were glycines (Fig. S3*A*). The docking model of TNT with NAD^+^ did not show any of these glycines in the direct vicinity to NAD^+^ (Fig. 1*B*). A disordered glycine-rich span was described in the Tse6 secretory particle, but no function was attributed (16). It is known that glycines provide conformational flexibility, which is often used in active sites of proteins to accommodate substrate binding (35) and inhibit aggregation (36). Thus, a role of some of those critical glycines is perhaps to provide additional flexibility to TNT. These glycines could contribute to the astonishing stability of TNT, which retains 50% of the activity at 95 °C (18). Indeed, we found a cluster of three frequently mutated glycines (Gly^813^, Gly^815^, and Gly^818^) directly below the essential arginine at position 780. Inspection of this “glycine cradle” (Fig. 5*E*) also points to its location directly below Arg^780^ of the QR*X*L motif. Our observation that the G818V mutation results in a highly unstable protein (Fig. S4, *C* and *D*) (18) supports the hypothesis that the glycine cradle plays an important role in the stability of TNT.

### Designed mutants define the catalytic site of TNT

Mutational analysis unexpectedly revealed that Phe^727^ was essential for TNT activity (Fig. 6). In the TNT–NAD^+^ docking model, Phe^727^ appears to form π-stacking interactions with the nicotinamide-ribosyl moiety of NAD^+^ (Fig. 5*F*). The role of Phe^727^ in aromatic scaffolding of NAD^+^ is supported by the structures of the NAD^+^ glycohydrolases Tne2 of *Pseudomonas protegens* and of Tse6 from *P. aeruginosa*, which were shown to have essential aromatic residues (Phe^330^ in Tne2 and Trp^344^ in Tse6) (21). The locations of these aromatic residues were almost identical to the location in of Phe^727^ in TNT, suggesting that this is a common structural feature of these NAD(P)^+^ glycohydrolases. Tne2 and Tse6 also contain glutamines (Gln^387^ and Gln^413^, respectively), which are similarly positioned to Gln^822^ in TNT (21). In TNT, Tyr^765^ is presumably also located in close proximity to the nicotinamide-ribosyl moiety of NAD^+^. Based on our finding that the Y765A mutant of TNT had a strongly reduced catalytic activity, we previously proposed that Tyr^765^ is involved in π-stacking interactions with NAD^+^ (18). However, the essentiality of Phe^727^ suggests that this phenylalanine provides the crucial π-stacking interactions with the nicotinamide-ribosyl moiety. The role of Tyr^765^ might instead be to provide hydrogen bonding with the 2′-OH of NAD^+^ (Fig. 5*F*). Interestingly, Phe^727^ is located immediately upstream of Pro^728^ and Pro^729^. Although we did not obtain TNT mutations at position 727 in our selection assay for nontoxic TNT mutants, we did obtain two mutants at position 728 (Fig. S3), indicating that Pro^728^ might stabilize Phe^727^ to participate in π-stacking interactions with NAD^+^.

Both D756A and R780A mutations rendered TNT unstable. Although the D756A mutant precipitated almost immediately upon cleavage from MBP, small amounts of the R780A protein could be purified, but ^1^H NMR spectra indicated that the protein was unfolded compared with WT, F727A, or R757A (Fig. 6*C*). Inspection of the structural model of TNT–NAD^+^ revealed that Asp^756^ and Arg^780^ are located at the deepest point of the NAD^+^-binding cleft of TNT. The carboxyl group and the guanidinium group are ∼2.1 Å apart and may form a salt bridge (Figs. 5*C* and 6*A*) connecting α-helix 3 and β-sheet 4 (Fig. 5*D*). Asp^756^ and Arg^780^ are part of the conserved DR*X*G and QR*X*L motifs (Fig. 5*D*), suggesting that this putative salt bridge is a conserved feature of TNT family members. In contrast, our observations that F727A and R757A were stable and structurally similar to WT TNT indicated that these residues do not contribute significantly to the intramolecular stability of TNT but rather represent true substrate-binding residues. Arginine residues in the catalytic site of other enzymes have been proposed to bind the NAD^+^ phosphate moieties (22), supporting our proposal that Arg^757^ of TNT is involved in NAD^+^ binding.

### Model for NAD^+^ binding by TNT

Based on our studies, we propose a model for NAD^+^ binding by TNT. In this model, essential interactions are the stabilization of NAD^+^ by Phe^727^ through π stacking interactions with the nicotinamide ring, whereas Tyr^765^ binds the ribose 2′-OH. The phosphate backbone of NAD^+^ is stabilized by Arg^757^. The salt bridge between Asp^756^ and Arg^780^ holds the active site together to enable hydrolysis of the nicotinamide-ribosyl bond. However, it is unclear which amino acid of TNT activates water to initiate the nucleophilic attack on the nicotinamide-ribosyl bond. It is conceivable that the flexibility of TNT enables a structural rearrangement that might translocate a distal residue closer to the nicotinamide-ribosyl bond. Further experiments are warranted to identify the general base needed to deprotonate water.

### How does TNT cleave NAD^+^?

Oppenheimer emphasized the importance of the ribose 2′-OH for stabilizing a putative oxocarbenium intermediate during NAD^+^ hydrolysis by multifunctional eukaryotic NADases (26). This is exemplified by the inability of eukaryotic NADases to cleave analogs substituted at the 2′-OH such as araF-NAD^+^. However, we observed slow cleavage of araF-NAD^+^ by TNT (Fig. 2*D*), suggesting that stabilization of the 2′-OH group does not play the predominant role hydrolysis of NAD^+^ by TNT. The mechanism of NAD^+^ hydrolysis has been established for multifunctional enzymes such as CD38 (37, 38) and for ADP-ribosyltransferases such as the cholera toxin (Table 1), whereas SPN of *S. pyogenes* is the only bacterial glycohydrolase that has been examined in mechanistic details. Cleavage of NAD^+^ by SPN was characterized as an ordered uni-bi-mechanism in which nicotinamide is released before ADP-ribose. This conclusion was based on the observation that ADP-ribose but not nicotinamide is a competitive inhibitor of NAD^+^ hydrolysis by SPN (13). By contrast, TNT is not inhibited by either nicotinamide or ADP-ribose and does not catalyze methanolysis of NAD^+^, preventing us from deducing further molecular details of NAD^+^ cleavage by TNT.

### Role of NAD^+^ and NADP^+^ hydrolysis by TNT for intracellular replication of M. tuberculosis

The catalytic activity of TNT is required for efficient replication of *M. tuberculosis* in macrophages (19). Several factors may contribute to the observed intracellular growth defect of *M. tuberculosis*, producing catalytically inactive TNT. An obvious explanation is that the initiation of necroptosis by TNT-dependent NAD^+^ depletion strongly reduces energy levels (19) and might disrupt phagosome maturation, membrane repair, and other cellular mechanisms required for control of *M. tuberculosis* growth in infected macrophages (39–42). An additional benefit of TNT-mediated NAD^+^ hydrolysis for *M. tuberculosis* could be the stimulation of NAD^+^ production by the NAD^+^ salvage pathway. It is known that recycling of NAD^+^ increases in *M. tuberculosis* during *in vivo* infection and under hypoxia (43). After translocation to the cytosol of infected macrophages TNT rapidly degrades NAD^+^ (19), which has been estimated to be present in low millimolar amounts in eukaryotic cells (44, 45), thus generating large quantities of nicotinamide. *M. tuberculosis* has been shown to take up nicotinamide, which is then converted into nicotinic acid by the nicotinamidase PncA and fed into the NAD biosynthesis pathway by PncB1 and PncB2, the two nicotinate phosphoribosyltransferases of *M. tuberculosis* (43). Studies using gene deletion mutants and specific inhibitors showed that interruption of NAD^+^ synthesis is bactericidal for growing cells of *M. tuberculosis*. Although the NAD^+^ salvage pathway is activated during infection of host cells, neither NAD^+^ recycling nor *de novo* synthesis is required for survival of *M. tuberculosis* in mice, indicating pathway redundancy (43). It is unclear whether NADP^+^ hydrolysis by TNT plays a role in the survival and/or replication of *M. tuberculosis* in macrophages. NADPH is the predominant form *in vivo* (46) and plays important roles in anabolic processes and in immune functions such as the oxidative burst generated in macrophages as an antimicrobial defense mechanism (46). However, TNT does not degrade NADPH directly as shown in this study, but it could prevent rapid regeneration from NADP^+^ by NADP^+^ reductases, which would eventually reduce NADPH levels and impact the activity of NADPH-utilizing enzymes. Thus, an additional function of TNT might be to reduce the oxidative burst and improve the survival of *M. tuberculosis* in infected macrophages (47). To distinguish between the roles of NAD^+^ or NADP^+^ hydrolysis by TNT for *M. tuberculosis in vivo*, it would be necessary to design a TNT mutant that only hydrolyzes either NAD^+^ or NADP^+^. However, all of the TNT mutants examined in this study had similar phenotypes for both NAD^+^ and NADP^+^ hydrolysis (Fig. 6*B*).

In conclusion, our findings characterize TNT as an NAD(P)^+^ glycohydrolase with properties distinct from other bacterial glycohydrolases. These differences may have evolved in the TNT domain to maintain a robust NAD(P)^+^ glycohydrolase activity within a much smaller enzyme to facilitate secretion by *M. tuberculosis* using the outer membrane protein CpnT by a currently unknown mechanism. Because the majority of the TNT residues that are involved in binding and/or hydrolysis of NAD^+^ are conserved in the TNT family, these findings may be important to understand the function of the over 300 TNT homologs.

## Experimental procedures

### Bacterial strains and reagents

*E. coli* strains DH5α and BL21 (DE3) were used for cloning and expression experiments, respectively, and were grown in LB broth or on agar with 0.5% glucose at 37 °C. 100 μg/ml carbenicillin and 25 μg/ml chloramphenicol were used for selection. Restriction enzymes were obtained from New England Biolabs. araF-NAD^+^ was obtained from Biolog. Other chemicals and reagents were purchased from Sigma–Aldrich and Difco.

### Expression and purification of recombinant TNT

All recombinant TNT plasmids were expressed in combination with the constitutive IFT expression plasmid pML1999 to prevent residual toxicity. *E. coli* BL21 (DE3) cells containing pML1999 and the T7 polymerase-based expression vector pML1995 encoding His_6_–MBP–TNT/IFT were inoculated in 50 ml of LB medium with 100 μg/ml carbenicillin, 25 μg/ml chloramphenicol, and 0.5% glucose to prevent expression of the target protein (uninduced sample). This starter culture was inoculated into 3 liters of the auto induction medium ZYP-5052 (48) supplemented with 100 μg/ml carbenicillin and 25 μg/ml chloramphenicol and was grown at one-fifth aeration in a 2-liter Erlenmeyer flask at 37 °C at 200 rpm for 24 h. The cells were harvested by centrifugation (at 6,000 × *g* at 4 °C for 15 min) and washed with 1× PBS (pH 7.4). The cell pellet was resuspended 1:5 in lysis buffer A (20 mm Tris-HCl, 300 mm NaCl, pH 7.4, supplemented with 1 mm PMSF, 2.5 units of benzonase per 10 ml of cell suspension and one tablet of a complete protease inhibitor mixture; Roche). The cells were sonicated for 1 min on/1 min off using Q55 Qsonica microprobe with a tip diameter of 6.35 mm at 50 watts on ice for five cycles. The cell lysate was clarified by centrifugation at 40,000 × *g* for 30 min. To remove IFT from the sample, either 8 m urea or 4 m guanidine-hydrochloride was added directly to the sample and incubated at room temperature on an end-over-end rotator until dissolved. The soluble fraction was then purified via Ni^2+^-NTA affinity chromatography (Thermo Scientific) under denaturing conditions involving three denaturing washes to remove IFT, followed by five washes using a buffer containing 25 mm sodium phosphates (pH 7.0), 150 mm NaCl, and 30 mm imidazole to refold the bound MBP–TNT on-column prior to elution. We did not find any overall difference with urea *versus* guanidine hydrochloride but found that the latter was faster and slightly more efficient in removal of IFT. We did not detect any differences in the final quality of enzyme by either method. The Ni_2_-NTA elutions were further purified by amylose resin (New England Biolabs), both according to the manufacturer's instructions. His_6_–MBP was removed via incubation with His_6_–TEV protease at a concentration of 1 μg of TEV protease per 100 μg of fusion protein at 25 °C for 24 h without shaking in 25 mm sodium phosphate (pH 7.0) and 150 mm NaCl. The protein was then incubated on HisPur cobalt resin (Thermo Scientific) to remove the His_6_–TEV protease and excess His_6_–MBP. The TNT protein was further purified on a size-exclusion column Superdex 75 (GE Healthcare). TNT samples were stored in 25 mm sodium phosphate (pH 7.0), 150 mm NaCl, and 50% glycerol at −20 °C.

### Site-directed mutagenesis of TNT

Mutations of the *tnt* gene in the expression plasmid pML1995 (18) were introduced by site-directed mutagenesis using standard overlap PCR using the primers listed in Table S1. Briefly, the TNT/IFT coding region was amplified from the parent vector with primers containing NdeI and HindIII restriction sites. Overlap PCR was performed to introduce the desired mutation. The final product was ligated back into the pML1995 parent backbone at 16 °C overnight or with pET21A backbone for constructs without MBP. 5 μl of the ligation mixture were transformed into *E. coli* DH5α by heat shock with SOC reconstitution for outgrowth and plated on LB medium containing carbenicillin with 0.5% glucose. Clones were analyzed by restriction digestion and sequencing to verify the mutations in the TNT coding region. The genes encoding nontoxic TNT mutants were subcloned into a pET21A His_6_–TEV (pML3928–31) vector without IFT.

### Purification of TNT mutants without MBP

TNT mutants that were amenable to purification without MBP were transformed into BL21 (DE3), and a single colony was grown overnight in 50 ml of LB medium and 1% glucose. The next day, the cell culture was used to inoculate 1 liter of ZYP5052 and further incubated for 8–10 h at 37 °C. The cells were then grown at 18 °C for 24 h, lysed, and purified via Ni_2_-NTA affinity chromatography. The buffer was exchanged using Amicon ultrafiltration tubes with a 3 kDa cutoff and the protein cleaved with TEV protease overnight at room temperature. Finally, the protein was run through a Ni_2_-NTA column to collect the flowthrough (TNT with no tags), followed by size-exclusion chromatography with a Superdex 75 column (GE Healthcare).

### End-point NAD^+^ glycohydrolase activity test

Preliminary analysis of NAD(P)^+^ hydrolysis activity was performed using the EnzyFluo NAD/NADH and EnzyFluo NADP/NADPH kit from Bioassay Systems following the manufacturer's recommendations. 100 ng of TNT or mutants were incubated with 100 μm of NAD(P)^+^ for 30 min at 37 °C prior to detection of residual NAD^+^. The data were analyzed on a BioTek Synergy Htx with Gen5 software. The data from the final read were normalized to the (−) TNT blank control and are represented as percentages of residual dinucleotide remaining in the well. Note that the detection of araF-NAD^+^ using the EnzyFluo Kit takes longer. This is likely due to the slower conversion of araF-NAD^+^ to araF-NADH by lactate dehydrogenase. For this reason, we incubated the reaction mixture for 4 h prior to detection. All experiments were repeated at least twice with identical results.

### Substrate hydrolysis by TNT measured by ^1^H NMR

NMR data were collected at 25 °C on a Bruker Avance II (700 MHz ^1^H) spectrometer equipped with a cryogenic triple-resonance probe and processed with Topspin. Substrate hydrolysis was measured by ^1^H NMR with excitation sculpting water suppression collected with eight scans and total recovery delay of 3.9 s. All experiments were conducted on a 500-μl reaction sample in a buffer containing 25 mm sodium phosphate (pH 7.0), 150 mm NaCl, 5% D_2_O in a final volume of 500 μl. 50 mm NAD^+^ and NADP^+^ stocks were prepared in the same buffer and frozen at −80 °C until use. Solutions were then diluted to 500 μm immediately prior to the experiment, and the actual concentration was determined based on the absorbance at 260 nm and the extinction coefficient for each dinucleotide. Baseline ^1^H NMR spectra were collected on the free substrate. An identical substrate sample was prepared in parallel in an Eppendorf tube, mixed with TNT, and placed into a clean NMR tube. The time of enzyme addition was recorded, and the spectra were collected automatically every 2 min until the substrate signal intensity had declined to the baseline level. Purified recombinant TNT was used at concentrations of 75 and 37.5 nm for the NAD^+^ and NADP^+^ hydrolysis experiments, respectively. Triplicate runs were also performed for the inhibition experiments using 0.5 mm nicotinamide, 0.5 mm ADP-ribose, and 2.5 mm ADP-ribose. Duplicate runs were performed for inhibition experiments using 2.5 mm nicotinamide. Signal assignments were obtained using standard correlation methods. A ^1^H NMR reference spectrum was recorded for 500 μm cyclic ADP-ribose in a buffer containing 25 mm sodium phosphate (pH 7.0) and 150 mm NaCl.

### Direct numerical solution of the Michaelis–Menten equation

The kinetics of an irreversible, uninhibited, enzymatically catalyzed reaction S + E → P + E, where S is a substrate, E is an enzyme, and P is a product, can be described by a Michaelis–Menten model,

(Eq. 1) $$-\frac{d\left[\text{S}\right]}{dt}=\frac{V_{\max}\left[\text{S}\right]}{K_{M}+\left[\text{S}\right]}$$

in which *V*_max_ is the maximum rate of conversion at given enzyme concentration, and *K_m_* is Michaelis constant. The reaction parameters *V*_max_ and *K_m_* are often obtained based on initial reaction rates determined for a series of reactions with varied substrate concentrations. The initial rates are then used in regression analysis of integrated form of Equation 1. However, the parameters *V*_max_ and *K_m_* can also be obtained more directly by analyzing the whole progress curve of a single reaction. In such case, parameters are calculated using nonlinear regression employing numerical solutions of various forms of Equation 1 (49). Also an explicit solution of Equation 1 based on Lambert omega function has been described that can be used in regression analysis of progress curves (50). Substrate and/or product concentrations during the reaction are monitored directly or indirectly, often by using spectroscopic methods. Monitoring a reaction by NMR spectroscopy is advantageous because concentrations of both substrate and product can be obtained directly and for the entire reaction course. Subsequent determination of *V*_max_ and *K_m_* from the progress curves can be achieved using either of the above outlined approaches (51). In our current work, progress curves were fitted directly with Equation 1 using nonlinear regression and differential equation solver available in Matlab. Well-resolved signals N-2, N-4, and N-6 of NAD^+^ and NADP^+^ were integrated for each kinetic series (as shown in Fig. 3), and the values were normalized by the integral in free NAD^+^ to correct for nonuniform intensities caused by relatively short relaxation period between NMR signal accumulations. Normalized integral values were multiplied by the NAD^+^ concentration and used as an input for fitting routine. The Matlab script is available upon request.

### Methanolysis of TNT

Methanolysis experiments were analyzed using reverse-phase HPLC. The reactions were as follows: 400 ng of TNT in a final volume of 1 ml in a buffer of 50 mm Tris (pH 7.4), 200 mm NaCl, with 5 mm NAD^+^ in either 10 or 20% methanol for 15 min at room temperature. For chemical methanolysis, NAD^+^ alone was incubated in 20% methanol for 90 min at 95 °C. Chromatography was performed on an Agilent 1260 Infinity HPLC using an Agilent Poroshell C18 4.6 × 50 mm column with 10 mm ammonium phosphate buffer (pH 5.5) and 2.5% acetonitrile. Isocratic elution was performed at 1 ml/min, and detection was performed at 260 nm.

### Reporter screen construction

The construction of the reporter screen was described previously (17).

### Molecular structures and sequence alignments

The representation of TNT in complex with NAD^+^ was rendered in PyMOL using a published docking model (18). Sequence alignments were obtained from the Pfam website for TNT (http://pfam.xfam.org/family/PF14021#tabview=tab7).^6^ The sequences were aligned using the T-Coffee server, and images were created using the ExPASy BoxShade tool. For Fig. 6, the Pfam seed file was put into Skylign and WebLogo, in which the occupancy and images were obtained and prepared, respectively.

## Author contributions

U. T., J. V., O. D., L. P. S. d. C., J. S. S., and M. N. conceptualization; U. T. validation; U. T., J. V., A. G.-G., D. W., and O. D. investigation; U. T., J. V., A. G.-G., and M. N. visualization; U. T., J. V., A. G.-G., and O. D. methodology; U. T., J. V., A. G.-G., and M. N. writing-original draft; U. T., J. V., O. D., L. P. S. d. C., J. S. S., and M. N. writing-review and editing; O. D., L. P. S. d. C., J. S. S., and M. N. supervision; M. N. funding acquisition; M. N. project administration.

## Supplementary Material

Supporting Information

## Footnotes

6 Please note that the JBC is not responsible for the long-term archiving and maintenance of this site or any other third party hosted site.

## Footnotes

5 The abbreviations used are:

TNT: tuberculosis necrotizing toxin
araF-NAD^+^: β-ara-2′-deoxy-2′-fluoro-nicotinamide adenine dinucleotide
MBP: maltose-binding protein
TEV: tobacco etch virus.
