# Supplementary material for: The tuberculosis necrotizing toxin is an NAD+ and NADP+ glycohydrolase with distinct enzymatic properties
Source: J Biol Chem. 2018 Dec 28;294(9):3024–36. doi: 10.1074/jbc.RA118.005832 (PMC6398120; doi:10.1074/jbc.RA118.005832)
Supplement: Supporting Information [file supp_294_9_3024__index.html]

The tuberculosis necrotizing toxin is an NAD+ and NADP+ glycohydrolase with distinct enzymatic properties — TNT glycohydrolase activity — Supporting Information 

# The tuberculosis necrotizing toxin is an NAD+ and NADP+ glycohydrolase with distinct enzymatic properties

## Supporting Information

- Supporting Information - The supplement contains 4 figures and 2 tables.
