# Supplementary material for: The tuberculosis necrotizing toxin is an NAD+ and NADP+ glycohydrolase with distinct enzymatic properties
Source: J Biol Chem. 2018 Dec 28;294(9):3024–36. doi: 10.1074/jbc.RA118.005832 (PMC6398120; doi:10.1074/jbc.RA118.005832)
Supplement: Supporting Information [file supp_RA118.005832_140743_1_supp_257947_pkctnw.pdf]

SUPPLEMENTARY INFORMATION

The tuberculosis necrotizing toxin is an NAD^+ and $NADP^+$ glycohydrolase with distinct enzymatic properties

Uday Tak[1], Jiri Vlach[1], Acely Garza-Garcia[2], Doreen William[1,$], Olga Danilchanka[1,&], Luiz Pedro Sório de Carvalho[2], Jamil S. Saad[1], and Michael Niederweis[1,*]

[1] Department of Microbiology, University of Alabama at Birmingham, AL, USA
[2] The Francis Crick Institute, London, UK
[$] Present Address: University of Rostock, Germany
[&] Present Address: Merck Exploratory Science Center, Cambridge, MA, USA

***To whom correspondence should be addressed:**
Michael Niederweis, Ph.D.
University of Alabama at Birmingham, Department of Microbiology, BBRB 609, 845 19th Street South, Birmingham, AL 35294
Phone: +1-205-975-4390, Email: mnieder@uab.edu

Supplementary Figures

```
TNT     1 ------------------------------------------------------------
Tse6    1 ------------------------------------------------------------
SPN     1 VSGKENKKSDVKYETTKVMEANATSSKEDNHVMHTLDGSMSTVWEENSPGGGVGEVLSYK
DTx     1 ------------------------------------------------------------
CD38    1 ------------------------------------------------------------

TNT     1 ------------------------------------------------------------
Tse6    1 ------------------------------------------------------------
SPN    61 FASPMRIGRILIVNGDTSSKENYYKKNRIAKADVKYYNGNKLVLFQKIELGDTYTKKPHH
DTx     1 ------------------------------------------------------------
CD38    1 ----------------------------------------------------MANCEFSP

TNT     1 -----------------------SHMRLSDEAVDPQYGEPLSRHWDFTDNPADRSRINPV
Tse6    1 ---------------------------------MGSSHHHHHHSQDPHDINYRGNRETAA
SPN   121 IEIDKKLDIDRIDIEVTEVHQGQNKDILALSEVTFGNMERDLFEKKFKEIKDKWVTDKQA
DTx     1 ------------------------------------------------------------
CD38    9 VSGDKPCCRLSRRAQLCLGVSILVLILVVVLAVVVPRWRQQWSGPGTTKRFPETVLARCV

TNT    38 VAQLMEDPNAPFGRD--PQGQPYTQERYQERFNSVGPWGQQYSNFPPNNGAVPGTRIAYT
Tse6   28 KFFKSKDIDPADAES--YMNGLDFNHPVRVETLAPGKNLWQY----------------QS
SPN   181 DEFIETADKYADKAVQMSAVASRAEYYRMYVSRKYQYKKEFVEKLKQVYKE-SGASHVTS
DTx     1 --GADDVVDSSKSFV--MENFSSYHGTKPGYVDSIQKGIQKPKSG---------------
CD38   69 KYTEIHPEMRHVDCQ--SVWDAFKGAFISKHPCNITEEDYQP----------------LM

TNT    96 -NLEKFLSDYG-PQLDRIGGDQGKYLAIM-------------------------------
Tse6   70 PGA-----------------PQGNWYTLSPRVQPTELGINPMGTNRAANTIE-PKVLNSY
SPN   240 KKDLMLAFDDAKRKSTIGRQENGLFVTSF-------------------------------
DTx    42 ------------TQGNYDDDWKGFYSTDN-----KYDAAGYSVDNENPLSGK-AGGVVKV
CD38  111 KLG-----------------TQTVPCNKILLWSRIKDLAHQFTQVQRDMFTLEDTLLGYL

TNT   123 EHGRPASWEQRALHVTSL----------------RDPYHAYTIDWL--------------
Tse6  112 RTTQK-------------------------------------------------------
SPN   269 -AEDMALLFTDQGKLKSADQIENIKGVDSGKYSDGVYQYEYDSE-------------LTK
DTx    84 TYPGLTKVLALKVDNAET----------------IKKELGLSLTEPLMEQVGTEEFIKRF
CD38  154 ADDLTWCGEFNTSKINYQ----------------SCPDWRKDCSNN--------------

TNT   153 --PEGWFIEV---------------SEVAPGCG------------QP---------GGSI
Tse6  117 -----VEVLR---------------STAAPTDDF-----------WSVKGQSYPAKGGAQ
SPN   315 NIDKLGYIRTASGDTPGANSLNIPGCQTWSGKH------------IE---------NSES
DTx   128 GDGASRVVLS---------------LPFA----------------EG---------SSSV
CD38  184 --PVSVFWKT---------------VSRRFAEAACDVVHVMLNGSRS---------KIFD

TNT   175 QVRI----------------------F------DHQN-----------------------
Tse6  146 QLFS--NEKGSFGLLPREGS----------------------------------------
SPN   354 ELIF----------------------P------SISVKDLKSKAVLAEIDAKGYFEIIDP
DTx   148 EYIN----------------------N------WEQA-----------------------
CD38  218 KNSTFGSVEVHNLQPEKVQTLEAWVIHGGREDSRDLCQDPTIKELESIISKRNIQFSCKN

TNT   184 EMRKVEELIRRGVLRQ--------------------------
Tse6      ------------------------------------------
SPN   386 TIIAPNGDHKKVTGRFKIKKMQDRMQDRK-------------
DTx   157 -----------KALSVELEINFETRGKRGQDAMYEYMAQACA
CD38  278 IYRPDKFLQCVKNPEDSSCTSEI-------------------
```

Fig. S1. TNT has limited homology to known NAD$^+$ hydrolyzing toxins

Alignment of *M. tuberculosis* TNT with the bacterial NAD$^+$ glycohydrolases Tse6 (*P. aeruginosa*) and SPN (*S. pyogenes*), the ADP-ribosyltransferase Dtx (Diphtheria toxin from *C. diphtheriae*) and the eukaryotic multifunctional NAD+ hydrolase/cyclase CD38. No significant homology was observed. The alignment was performed using the Tcoffee server and boxshade tools.

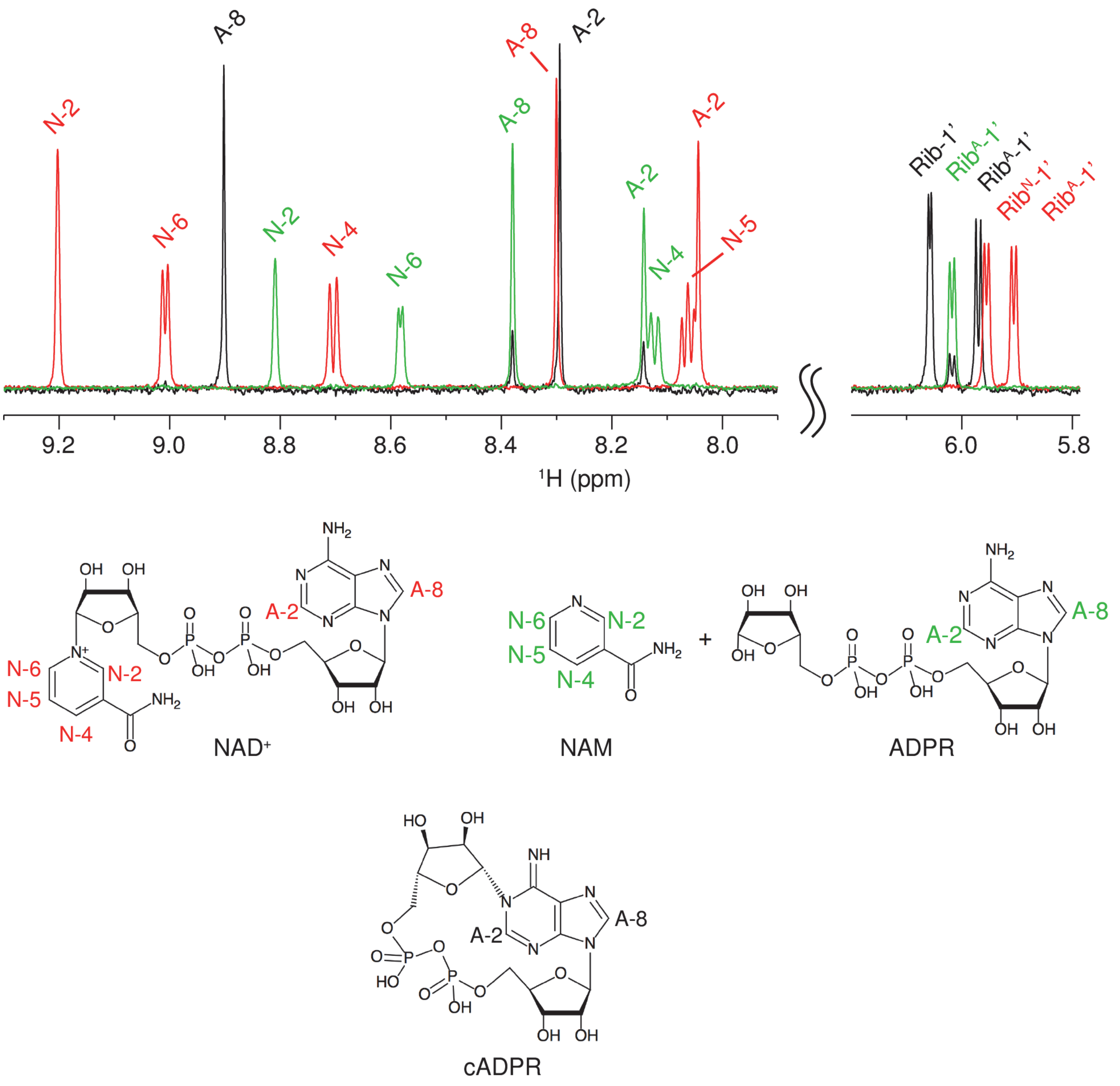

Fig. S2. Nicotinamide and ADP-ribose are the only products of TNT-mediated NAD^+ hydrolysis
Aromatic and anomeric regions of ^1H NMR spectra of NAD^+ (red), purified cyclic-ADP-ribose (black) and hydrolysis products of 500 μM NAD^+ after incubation with 75 nM TNT for 30 minutes (green). Signals assignments to structures shown below are indicated. The assignments of cADPR signals are reported elsewhere (1). The commercially obtained cADPR contained small amount of ADPR.

A

Mutation	Frequency	Mutation	Frequency	Mutation	Frequency	Mutation	Frequency
A656V	1	G732C	3	G772V	1	A811E	1
G662D	1	A733V	1	P774L	2	A811V	1
W669C	1	G736V	1	P774T	1	G813E	2
L688H	1	G752V	1	A775E	2	G813V	1
A694D	3	D756Y	1	W777R	1	G813W	1
P695Q	1	R757L	2	W777L	1	G815V	3
G697S	1	R757S	1	R780L	1	G815D	4
G697C	2	I758M	1	R780S	3	G815C	2
G697V	2	G759V	1	A781D	2	Q816K	2
G702V	1	G763S	3	A781V	1	G818V	9
G718C	1	G763V	1	H783Q	1	G819R	1
P728Q	4	G763C	2	V784L	2	Q822K	2
P728L	2	A767E	4	P800H	1	L844S	1
G732V	2	G772W	1	G802V	1		

B

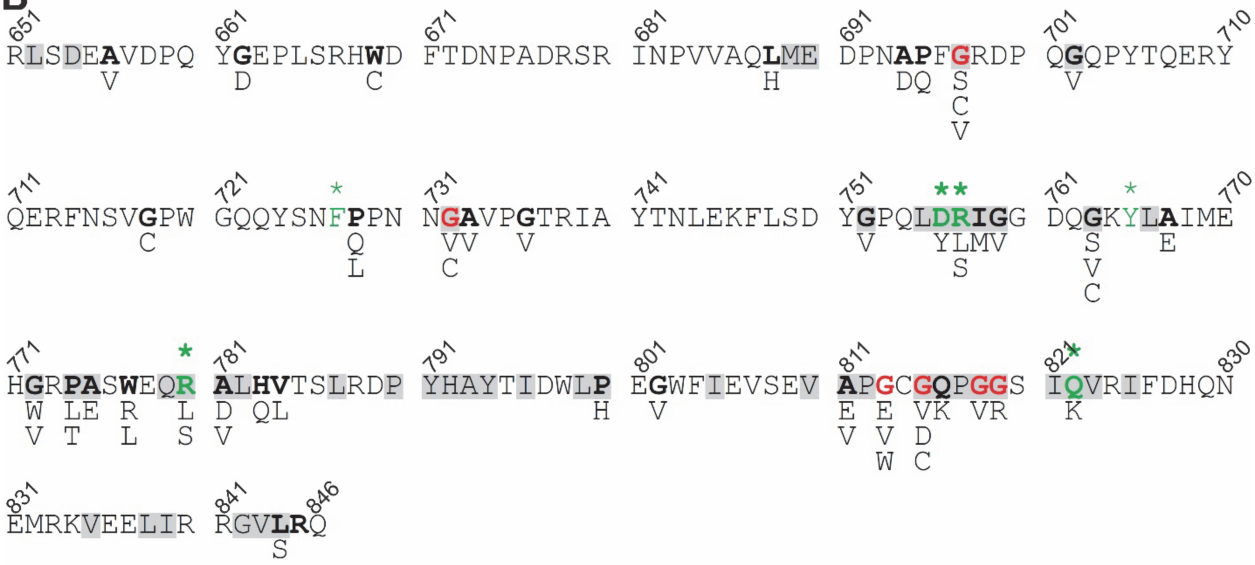

Fig. S3. TNT mutations obtained by selection for non-toxic mutants in *E. coli*.
A. Type and frequency of mutations obtained by selection for non-toxic TNT mutants in *E. coli***.**
B. Sequence map of mutations. The N-terminus of TNT was chosen as defined previously based on the shortest deletion construct which retained full toxicity (2). The numbering is based on the CpnT amino acid sequence. Residues which are conserved in more than 70% of the members of the TNT family are shown with a grey background. Residues mutated in the selection assay are shown in bold. Catalytically important residues are shown in green, while mutated glycines are red.

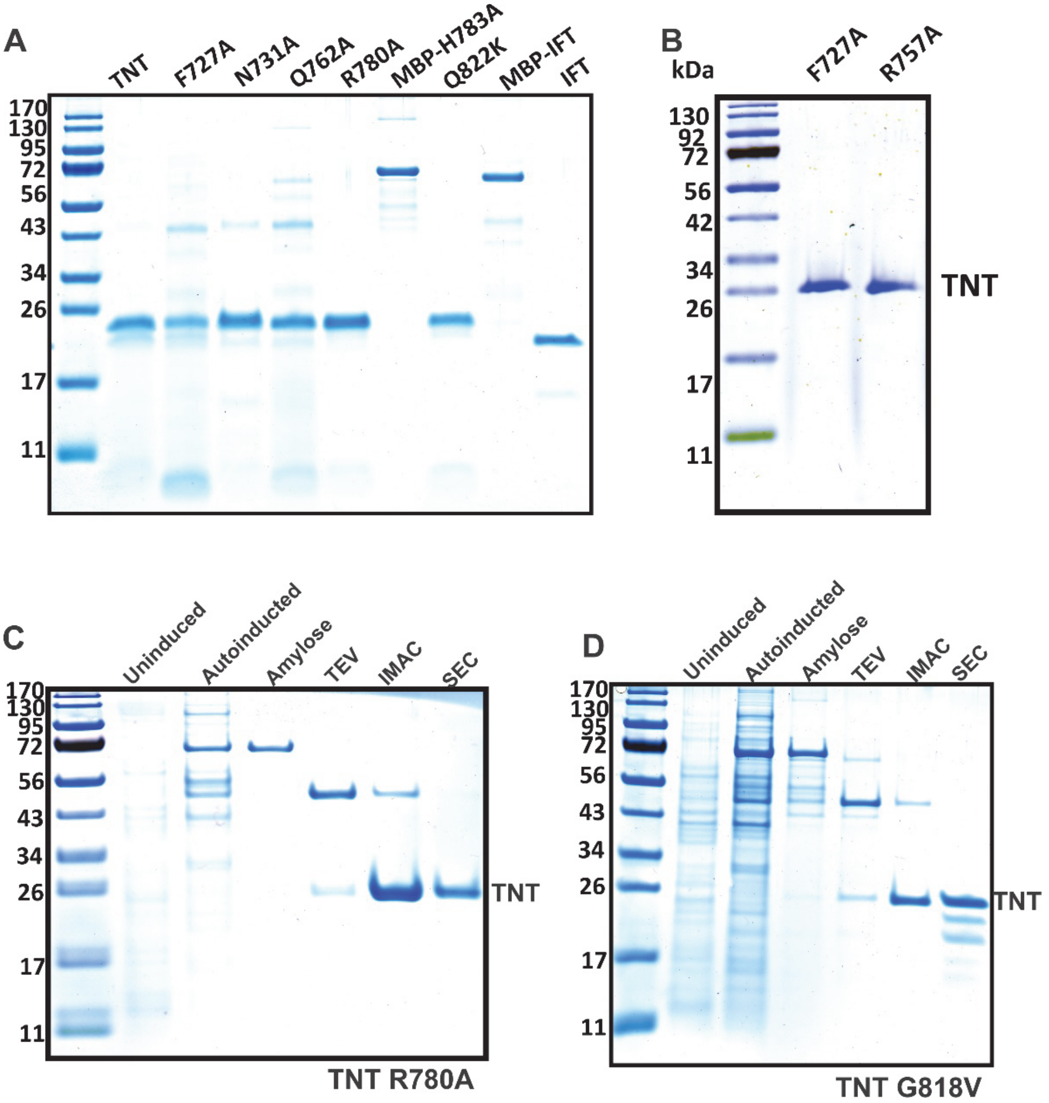

Fig. S4. Purity of TNT mutant proteins

A. Colloidal Coomassie stained denaturing polyacrylamide gel indicating the purity of proteins used in this study. TNT Mutants were purified via the denaturing protocol indicated in materials and methods.

B. Purity of TNT mutants obtained using a His_6TEV-TNT construct using nickel affinity chromatography, TEV cleavage, nickel affinity chromatography, and size exclusion on a Superdex 75 for non-toxic mutants.

C. Representative gel showing purification of TNT mutants using a His_6-TEV-MBP-TNT fusion construct.

D. Representative gel showing purification of TNT-G818V mutant using a His_6-TEV-MBP-TNT fusion construct. Degradation is apparent following size exclusion, as indicated by the presence of lower molecular weight bands compared to TNT R780A in panel C.

Supplementary Tables

Primer	Sequence (5’->3’)	Reference
TNT Fwd	CAGAGCCATATGCGGTTATC	This Work
IFT Rev	GCAAGCTTTAACCCTTATAGTCCTTCCAAA	This Work
TNT Rev	ATTAAAGCTTAATCACTGTCGCAACACCCCGCGCC	This Work
TNT F727A Fwd	CAGTACTCTAATGCTCCGCCTAACAATGG	This Work
TNT F727A Rev	CCATTGTTAGGCGGAGCATTAGAGTACTG	This Work
TNT N731A Fwd	CTAATTTTCCGCCTAACGCTGGTGCGGTTC	This Work
TNT N731A Rev	GAACCGCACCAGCGTTAGGCGGAAAATTAG	This Work
TNT D756A Fwd	GGCCCCCAGCTAGCTCGTATAGGCGGCGAT	This Work
TNT D756A Rev	ATCGCCGCCTATACGAGCTAGCTGGGGGCC	This Work
TNT R757A Fwd	CCCAGCTAGATGCGATAGGCGGCGATC	This Work
TNT R757A Rev	GATCGCCGCCTATCGCATCTAGCTGGG	This Work
TNT Q762A Fwd	CGTATAGGCGGCGATGCGGGCAAGTACCTGG	This Work
TNT Q762A Rev	CCAGGTACTTGCCCGCATCGCCGCCTATACG	This Work
TNT R780A Fwd	ATCATGGGAACAAGCTGCCCTGCACGTGACG	This Work
TNT R780A Rev	CGTCACGTGCAGGGCAGCTTGTTCCCATGAT	This Work
TNT H783A Fwd	CAACGTGCCCTGGCCGTGACGTCGTTA	This Work
TNT H783A Rev	TAACGACGTCACGGCCAGGGCACGTTG	This Work
TNT H792N Fwd	CGCGACCCCTACAACGCGTATACCATT	This Work
TNT H792N Rev	AATGGTATACGCGTTGTAGGGGTCGCG	This Work

Table S1: Oligonucleotides used in this study

Plasmid	Description	Reference
pML1995	pML1970 derivative, pBR322 origin, *bla, lacI*^{q}, p$_{T7lac}$ His6-*malE*-polyN-TEV-*tnt ift*	(3)
pML1999	pDP3615 derivative, P15A origin, *cam,* p$_{tet}$-*ift*	(3)
pML1974	pML1970 derivative, pBR322 origin, *bla, lacI*^{q}, p$_{T7lac}$ His6-*malE*-polyN-TEV *ift*	(3)
pML2123	ColE1 origin, *hyg,* oriM, p$_{smyc}$-*tnt-HA-gfp*	(2)
pML3901	pML1995 derivative, pBR322 origin, *bla, lacI*^{q}, p$_{T7lac}$ His6-*malE*-polyN-TEV-*tnt*$_{F727A}$ *ift*	This Work
pML3902	pML1995 derivative, pBR322 origin, *bla, lacI*^{q}, p$_{T7lac}$ His6-*malE*-polyN-TEV-*tnt*$_{N731A}$ *ift*	This Work
pML3903	pML1995 derivative, pBR322 origin, *bla, lacI*^{q}, p$_{T7lac}$ His6-*malE*-polyN-TEV-*tnt*$_{Q762A}$ *ift*	This Work
pML3904	pML1995 derivative, pBR322 origin, *bla, lacI*^{q}, p$_{T7lac}$ His6-*malE*-polyN-TEV-*tnt*$_{R780A}$ *ift*	This Work
pML3906	pML1995 derivative, pBR322 origin, *bla, lacI*^{q}, p$_{T7lac}$ His6-*malE*-polyN-TEV-*tnt*$_{H783A}$ *ift*	This Work
pML3907	pML1995 derivative, pBR322 origin, *bla, lacI*^{q}, p$_{T7lac}$ His6-*malE*-polyN-TEV-*tnt*$_{H792N}$ *ift*	This Work
pML3909	pML1995 derivative, pBR322 origin, *bla, lacI*^{q}, p$_{T7lac}$ His6-*malE*-polyN-TEV-*tnt*$_{G818V}$ *ift*	This Work
pML3910	pML1995 derivative, pBR322 origin, *bla, lacI*^{q}, p$_{T7lac}$ His6-*malE*-polyN-TEV-*tnt*$_{Q822K}$ *ift*	This Work
pML3914	pML1995 derivative, pBR322 origin, *bla, lacI*^{q}, p$_{T7lac}$ His6-*malE*-polyN-TEV-*tnt*$_{R780A}$	This Work
pML3916	pML1995 derivative, pBR322 origin, *bla, lacI*^{q}, p$_{T7lac}$ His6-*malE*-polyN-TEV-*tnt*$_{H783A}$	This Work
pML3919	pML1995 derivative, pBR322 origin, *bla, lacI*^{q}, p$_{T7lac}$ His6-*malE*-polyN-TEV-*tnt*$_{G818V}$	This Work
pML3920	pML1995 derivative, pBR322 origin, *bla, lacI*^{q}, p$_{T7lac}$ His6-*malE*-polyN-TEV-*tnt*$_{Q822K}$	This Work
pML3922	pML1995 derivative, pBR322 origin, *bla, lacI*^{q}, p$_{T7lac}$ His6-*malE*-polyN-TEV-*tnt*$_{R757A}$ IFT	This Work
pML3923	pML1995 derivative, pBR322 origin, *bla, lacI*^{q}, p$_{T7lac}$ His6-*malE*-polyN-TEV-TNT$_{D756A}$ IFT	This Work
pML3928	pML1995 derivative, pBR322 origin, *bla, lacI*^{q}, p$_{T7lac}$ His6-*malE*-polyN-TEV-*tnt*$_{F727A}$	This Work
pML3929	pML1995 derivative, pBR322 origin, *bla, lacI*^{q}, p$_{T7lac}$ His6-*malE*-polyN-TEV-*tnt*$_{R757A}$	This Work
pML3930	pML1995 derivative, pBR322 origin, *bla, lacI*^{q}, p$_{T7lac}$ His6-*malE*-polyN-TEV-*tnt*$_{R780A}$	This Work

Table S2: Plasmids used in this study. Derivative indicates the parent plasmid which was used to construct the respective plasmid. Origin stands for origin of replication. The *bla, hyg,* and *cat* genes confer resistance to ampicillin, hygromycin, and chloramphenicol, respectively. *MalE* encodes the maltose-binding protein of *E. coli*. TEV is the abbreviation for the cleavage site of the protease from the Tobacco Etch virus. Tet stands for tetracycline.

SUPPLEMENTARY REFERENCES

1. Walseth, T. F., and Lee, H. C. (1993) Synthesis and characterization of antagonists of cyclic-ADP-ribose-induced Ca2+ release, *Biochim Biophys Acta* **1178**, 235-242
2. Danilchanka, O., Sun, J., Pavlenok, M., Maueroder, C., Speer, A., Siroy, A., Marrero, J., Trujillo, C., Mayhew, D. L., Doornbos, K. S., Munoz, L. E., Herrmann, M., Ehrt, S., Berens, C., and Niederweis, M. (2014) An outer membrane channel protein of *Mycobacterium tuberculosis* with exotoxin activity, *Proc Natl Acad Sci U S A* **111**, 6750-6755
3. Sun, J., Siroy, A., Lokareddy, R. K., Speer, A., Doornbos, K. S., Cingolani, G., and Niederweis, M. (2015) The tuberculosis necrotizing toxin kills macrophages by hydrolyzing NAD, *Nat Struct Mol Biol* **22**, 672-678
